# Meeting report of the Mosquito Kolymbari Meeting 2013

**DOI:** 10.1179/2047772413Z.000000000163

**Published:** 2013-12

**Authors:** Michael Povelones, George K Christophides

**Affiliations:** 1 Imperial College London, UK; 2 University of Pennsylvania, Philadelphia, PA, USA

On 15–19 July 2013 the European Molecular Biology Organization (EMBO) and the Orthodox Academy of Crete, in Kolymbari, Chania, Greece, hosted the 6th conference on the Molecular and Population Biology of Mosquitoes and Other Disease Vectors. These vectors transmit serious parasitic and viral diseases, the most devastating of which are malaria and dengue, which together cause over 300 million cases, kill over one million people every year, and threaten half of the world’s population.

This conference series was initiated in 2003 soon after completion of the genome sequencing of the mosquito *Anopheles gambiae*, the major malaria vector in sub-Saharan Africa, in order to create a forum for dissemination of novel and unpublished scientific data toward molecular and functional genomic analyses. Since that initial event, the meeting is repeated every two years and is now established as one of the most important meetings and a great tradition in the field. It brings together vector biologists focusing on a wide spectrum of topics from basic biology to disease transmission and control. The principal theme of the current meeting was integrating data originating from basic biological research towards disease control. In addition to EMBO, the other major funder was the Wellcome Trust. Both EMBO and Wellcome Trust funds mainly supported participants from disease endemic and medium to low income countries through travel awards. This was the fifth time EMBO funded the conference (only the 2011 event was not funded by EMBO) and the first time for the Wellcome Trust. The European Infrastructure Project INFRAVEC, *Pathogens and Global Health,* and Imperial College London also sponsored the meeting in diverse ways.

**Figure 1:**
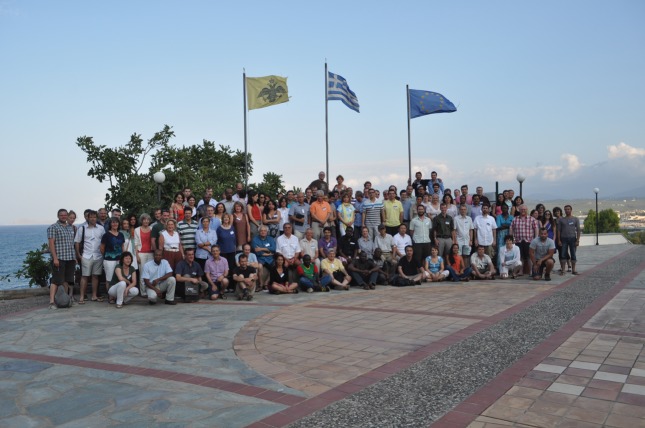
Mosquito Kolymbari Meeting 2013 participants in front of the conference building of the Orthodox Academy of Crete

The Scientific Organizing Committee consisted of experts in the field from across the globe and was chaired by George Christophides. The main organizers of this event were, apart from the Chair of the OC, Anthony A. James of the University of California Irvine, John Vontas of the University of Crete and Michael Povelones. The OC selected 150 presentations from over 260 submitted abstracts to make a scientific programme covering nine different subject areas spanning most aspects of vector biology in which molecular and population research has had a big impact in recent years, while the concluding session emphasized examples of innovation and translation (Fig. 1). The meeting was opened by a plenary speaker and each session was introduced by thematic keynote speakers. Apart from the many presentations and the keynote speakers, a new feature of this meeting compared to previous in the series were sessions of 5-minute turbo talks during which presenters revealed the main messages of their posters. Abstracts for all presented material are provided in this special edition of *Pathogens & Global Health*. The purpose of this report is to provide some highlights of work presented at the meeting.

The keynote speaker who kicked-off the meeting, Kevin Baird from Eijkman-Oxford Clinical Research Unit in Jakarta, discussed the epidemiology and global health threat of *Plasmodium vivax*. He made the argument that the widespread assumption that vivax malaria is less pernicious than *Plasmodium falciparum* malaria is inaccurate. Approximately 2.8 billion people live in areas at risk of infection by *P. vivax*. Its epidemiology is complicated by the fact that the dormant hypnozoite liver stage is practically undetected and does not respond to treatment with blood stage antimalarials. The use of primaquine in the anti-relapse treatment of *P. vivax* malaria is compromised by its propensity to cause acute haemolytic anaemia in individuals with glucose-6-phosphate-dehydrogenase deficiency. For further details, see the accompanying paper in this issue.

Session 1 on Host/Pathogen Interactions and Immunity was introduced by Frank Jiggins, who presented his group′s exciting work on investigating the genetic basis of disease susceptibility and transmission in the mosquito *Aedes aegypti*. They used a variety of techniques including genome sequencing and exome re-sequencing of natural as well as laboratory populations to dissect the mechanisms underlying their differences in susceptibility to pathogens. The presentation sparked lively discussions about the effectiveness of new technologies in discovering disease related traits in natural populations and why some of these traits seemed to be controlled by single loci. Additional speakers in this session also considered genomics technologies such as re-sequencing and SNPchips to study the interactions between hosts and pathogens, while most speakers focused more on molecular interactions between mosquito vectors and their pathogens using functional genetics and biochemical analyses. Louis Lambrechts presented data using field caught strains of *Ae. aegypti* from Thailand and different isolates of Dengue virus to show that the mosquito genotype is important for the outcome of infection, but it also depends on the virus strain. Cheng-Chen Chen presented data showing that the cellular recycling and homeostatic process of autophagy may also function to protect *Ae. aegypti* from infection by Dengue virus, bringing the very topical field of autophagy into the vector research agenda. In her talk, Dina Vlachou demonstrated how transcriptomic analyses can be used to simultaneously analyse the responses of both the host and the pathogens, using *An. gambiae* infected with field isolates of *P. falciparum*. This work followed by silencing of genes in mosquitoes and disruption of genes in the parasites revealed a battery of novel regulators of malaria transmission.

Fernando Noriega kicked off Session 2, Physiology and Development, discussing the production of Juvenile Hormone (JH). JH production is very dynamic and its production is controlled in different phases in the mosquito including at eclosion and in response to nutrient sensing. It is important to understand the production of this key hormone because it regulates follicular development and ultimately controls the reproductive capacity of the mosquito. Flaminia Catteruccia discussed a protein identified in mated female *An. gambiae* called Male Induced Stimulator of Oogenesis (MISO) that is responsible for the increase in egg production by mated female mosquitoes compared to control virgin females. Potential signals required for the production of MISO following mosquito mating were discussed. In the same session, Katia Gondim and Pedro Oliveira focused on the acquisition of lipids and proteins, respectively, by the insect vector of Chagas disease, *Rhodnius prolixus*. It was revealed that kissing-bugs distribute to fat body, muscles, and ovaries approximately double the amount of lipids they consume in a blood meal so they must synthesize their own, presumably from acetylCoA synthesized from digested proteins. Pedro Oliveira discussed an RNAi screen to examine the role of redox in innate immunity, digestion, and ovary/egg production.

There were two talks on ticks in Session 2. The first by Michail Kotsyfakis described a component of the tick saliva, the serine protease inhibitor IRS-2, that is important for blood feeding by preventing platelet aggregation, and discussed the role of saliva components in the spread of bacteria causing anaplasmosis and Lyme disease. The second by Petr Kopáček provided insights into the importance of heme/iron metabolism in tick feeding. Ticks strongly upregulate genes involved in hemoglobin digestion and the prospect of interfering with this process as a control measure was discussed.

Session 3 on Population Genetics began with a keynote by Ken Vernick discussing genetic differences in the field in *Anopheles* species that affect malaria transmission. Of the seven *An. gambiae* sister species, *An. gambiae sensu stricto* and *An. arabiensis* are the most important malaria vectors. The focus of this talk was the *An. gambiae* M and S forms from field populations in Burkina Faso. Because M form mosquitoes are known to contain loci exhibiting loss of genetic diversity it is assumed that they have recently undergone selection. The hypothesis presented was that other genes showing little diversity in M form mosquitoes may reveal novel components of the innate immune system and that the alleles that were selected in M form mosquitoes are also present in S form. A number of candidates were tested by RNAi in M form mosquitoes and shown to influence the outcome of *Plasmodium* infection. Alessandra della Torre extended the discussion of M and S form mosquitoes and presented historical data and the rationale that has led to the recent designation of these forms as separate species. The evidence includes lack of gene flow between M and S in analysis of over 400k SNPs. The underlying cause may be ecological barriers to M and S mating. On the other hand, evidence against M and S being separate species is that M and S hybrids occur in part of the *An. gambiae* range and that hybrids generated in the laboratory are viable and fertile. Nevertheless, the consensus is that these forms should now be considered as separate species and thus, the M form has been given the name *An. coluzzii*. There is very strong data to consider these as distinct species despite data showing that fertile hybrids can be formed in the laboratory and also evidence for gene flow between the two in the field. In support of this, it was noted that many of the presenters at the meeting had already replaced the species name *An. gambiae* M form with *An. coluzzi*. It has been recognized by most participants that after many years of constructive and vibrant discussions, this first part of the work that was initiated by Mario Coluzzi and dominated the field of mosquito genetics for the better part of the last two decades is now approaching its closing stages.

In the same session Bill Black presented the population structure of *Ae. aegypti sensu latu* in Senegal. Two major clades were identified to be distinct and found to be present in both previously identified subspecies, *Ae. aegypti formosa* and *Ae. aegypti aegypti*. Different chromosomal forms were described with the message that *Ae. aegypti* population structure is likely more complex than previously appreciated. A talk by Giuliano Gasperi explored the spread of *Ae. albopictus* throughout the world. He showed strong evidence for the role in human activity in the spread of this important emerging disease vector. It was further revealed that the spread of *Ae. albopictus* occurred chaotically rather than in a step-by-step migration.

Session 4, Symbionts, Microbiota and Immunity, started with a keynote from Serap Aksoy who discussed her laboratory’s research on the obligate endosymbiont of tsetse flies, *Wigglesworthia*. These bacteria make key nutrients, without which female flies are sterile. Not only do these bacteria play a role in the reproductive physiology but they also prime the immune system of tsetse and increase their resistance to other pathogens. They found that cells express *PGRP-LB* in proportion to the density of *Wigglesworthia* they harbor. Without this endogenous signal, flies produce less *PGRP-LB* and are more susceptible to other pathogens. There were five talks concerning how *Wolbachia* influence disease transmission. It was clear at the meeting in 2011 that one important goal for the field was stable *Wolbachia* infection of *Anopheles* and it was reported here by Zhiyong Xi, whose lab managed to achieve stable infection and transmission of *Wolbachia* in *An. stephensi*. As with previously reported transient infection, mosquitoes stably infected with *Wolbachia* are less susceptible to infection by *Plasmodium*. It will be interesting to follow whether this milestone leads to *Wolbachia*-based malaria control measures like those currently being field-tested for *Ae. aegypti* to control Dengue transmission. Another emerging area described in work presented by Marcelo Jacobs-Lorena is the engineering of natural symbiotic midgut bacteria to produce anti-malarial compounds. They have started testing genetically modified *Pantoea agglomerans* strains producing five different effector molecules. The best inhibitor reduced *Plasmodium* infection levels by 98% and decreased by 84% the proportion of infected mosquitoes.

Session 5 on Genomics and Bioinformatics was introduced with a keynote by Dominic Kwiatkowski. He provided insights that could only be revealed by deep sequencing of *Plasmodium* field isolates after describing the technical hurdles in working with AT-rich samples that are contaminated with human DNA. They have identified 400k credible SNPs out of a pool of 2 million and have incorporated 2 terabases of data into a population genetics database. Using these data they have found that in Africa no two samples are the same and that there are 250k singleton variants exhibiting geographic patterns which indicate population expansion. Interestingly, in contrast there are fewer rare variants in samples from Cambodia indicating a population bottleneck, which may be due to drug resistance. Furthermore, a SNP in these Cambodian populations was present in a gene for DNA mismatch repair raising the hypothesis that these populations might be more prone to mutation. The lessons learnt from the *Plasmodium* work provide the framework for a current effort to sequence 1000 anopheline genomes covering samples from 11 countries and over 30 locations. Complementing the keynote, Dan Neafsey gave an update on the 16 *Anopheles* genomes project. Given the scale of participation and the widespread response from the community it is clear that these projects will provide many exciting insights at the next Kolymbari meeting. Zhijian Tu discussed progress made in largely unchartered territory, the Y chromosome of *An. gambiae* and *An. stephensi*. His lab has been trying to identify genes on the Y chromosome by comparing the ratio of female to male alignments. This led to the discovery of seven candidate genes on the Y including a homolog of a human Y-linked transcription factor and a gene named GUY1. This gene is transcribed 2-3 hours after fertilization and is the earliest known expressed Y gene. A luciferase reporter was active in both males and females, and the function of GUY1 is currently being analysed using RNAi. As in previous meetings of the series, VectorBase, the home of vector genome sequences and other genomic resources was represented by talks from Dan Lawson and Scott Emrich, on genome annotation and the new database structure and interface, respectively. Additional talks about specific aspects of VectorBase were presented in other sessions including population genetics and insecticide resistance by Bob MacCallum and Emmanuel Dialynas, respectively.

The keynote for Session 6 on Insecticides and Resistance was given by Mylene Weill and she discussed molecular and evolutionary mechanisms of mosquito resistance to organophosphate. She described insights obtained from sampling performed in Montpellier during years of insecticide treatment. It was observed that the evolution of resistance is fast and complex with migration of resistant mosquitoes playing a crucial role in both parameters. Resistance is also evolutionarily costly and without active insecticide pressure, resistance alleles are lost from the population as they incur a fitness disadvantage. She is currently using this data to model insecticide resistance to help plan campaigns for the sustainable use of extant and newly developed insecticides. Janet Hemingway described the development of a decision making tool to help spray teams use the right spray at the right place and time. This tool integrates complex data sets in an easy to use package. Diagnostic kits to measure resistance were developed to assist in operational control decisions. It is important to closely manage the use of insecticides as the next new compound is projected to be available in 2017. Another interesting theme in this session touched upon by Jean-Philippe David and Vasileia Balabanidou was the influence of insecticide resistance on vector immunity.

Session 7 covered Genetically Modified Vectors. Talks in this session addressed basic biological questions as well as technical advances in transgenic technologies, such as those described in the keynote by David O’Brochta. He described improvements they have made in enhancing the mobility of transposable elements in *Anopheles*. Overall, they are now able to achieve a transposition rate of 6% with a slight female bias. This technology is now being explored to develop a gene-capture system where the transposable element will contain a fluorescent reporter that will splice into endogenous transcripts. To maximize the use of new lines Gal4 transcriptional activator is being incorporated into the transposition system. The expectation is that these Gal4 driver lines will be available to the community to drive gene expression under the control of UAS sites in tissues of interest or at particular stages of development as is done routinely in *Drosophila*. Possibilities for using this technology could be expression of anti-plasmodial factors, fluorescent reporters or expression of dsRNA for gene knockdown. In this session, Andrea Crisanti presented the European Infrastructure project INFRAVEC and he described to the community how this distributed infrastructure can be accessed through submission of research proposals to generate transgenic mosquitoes, to carry out high throughput sequencing and have access to bioinformatics expertise. An article on INFRAVEC and its activities can be found elsewhere in the issue.

Maureen Coetzee gave the keynote for Session 8, Ecology, Epidemiology and Behaviour. She discussed her studies of the African malaria mosquito *An. funestus*. Members of this vector species form two main clades within which there is a lot of diversity. The distribution of these clades and their impact on parasite transmission and insecticide resistance are topics that are currently being addressed. This talk provided the groundwork for other presentations in the session looking at genetic differences in mosquito populations with different behaviours including predator avoidance in larval pools, host preference for blood meal, mating swarm formation, and mate selection in swarms. Using behavioural differences to map genes associated was a common theme throughout this session and was nicely illustrated in a talk by Carolyn McBride. She presented work studying the differences in host seeking behaviour of two populations of *Ae. aegypti* from the same area in Rabai, Kenya. One population bred in tree holes in the forest and prefers rodent hosts, whereas the other domestic population bred in water containers in the village. Host preference was maintained in laboratory stocks of these lines and was used as an assay to identify the genes involved. Among these, AaegOR4 encoding an odorant receptor was found to have 8 major haplotypes, with up to 24 amino acid differences. Sulcatone was identified as the ligand for AaegOR4. Sulcatone is produced by humans, but not rodents and was found to attract more domestic mosquitoes than forest mosquitoes. There were two talks in this session by Tom Burkot and Greg Madey discussing the Vector Ecology and Control Network, VECNet. The purpose of this network is to utilize spatially explicit information to model the impact of interventions on malaria transmission towards guiding the implementation of control and eradication measures.

Session 9, Innovation and Translation, the last session of the meeting, featured different strategies for moving from the laboratory to the field. Scott O’Neil gave the keynote and talked about the Eliminate Dengue program. This program deploys *Wolbachia* infected *Ae. aegypti* to block the spread of the virus. This strategy has attracted a lot of attention because of the ability of the *Wolbachia* to spread in the population. Infected females pass on the bacteria to all their progeny regardless of the infection status of their partner. Infected males are able to successfully mate with infected females, but importantly when they mate with uninfected wild females the resulting eggs do not hatch. Since infected females can mate with wild males and transmit *Wolbachia* to nearly 100% of their offspring and infected males blunt the reproductive success of wild females, *Wolbachia*, once seeded can spread through the population. It was noted that *Wolbachia* are naturally present in many insect species with no known harmful effects towards humans or other animals. Nevertheless, when outlining the overall considerations to implement a release programme, Scott stressed the crucial role of community engagement.

Luke Alphey provided an update on mosquito population control efforts by the company Oxitech, where he is the Chief Scientific Officer. Oxitech is using the Release of Insects Containing a Dominant Lethal (RIDL) strategy, where transgenic mosquitoes are released. Luke discussed release of *Ae. aegypti* OX513A males. These mosquitoes can mate competitively with wild females resulting in progeny that die late in larval development. Ongoing field trials in Grand Cayman and Brazil have shown initial population reductions of 60–80%. The session also included some out-of-the-box ideas for developing new tools to prevent disease transmission.

Barry Beaty described using nanoparticles as molecular mosquitocides. He discussed the efficacy and targets of different shapes and sizes of nanoparticles and also the search for an environmental uptake and delivery system. Anthony A. James provided a cautionary tale on transitioning from the laboratory to the field. The work he described showed that although there was a large correspondence between the transcriptional responses of different *Ae. aegypti* strains to blood feeding, there are also prominent differences. Adding to the complexity, mosquitoes also exhibit non-overlapping transcriptional profiles to different Dengue serotypes. They are now focusing on identifying a universal set of infection-responsive for all combinations of *Ae. aegypti* strains and Dengue serotypes. Stephen Torr discussed new implementations of tsetse fly control measures that they have developed. Previous traps were large black boards impregnated with insecticide and mimicking the silhouette of a cow, the preferred blood meal host for the fly. Though effective, these lures were cumbersome to deliver and maintain in the field. By studying the biological cues attracting tsetse to the traps they were able to significantly reduce the size of the traps to a small piece of blue fabric. These are relatively easy to distribute and the population reduction achieved is in line with models. George Dimopoulos presented the portfolio of his research that aims to target the vectors and their disease transmission capacity. Finally, George Christophides reported the successful completion of the flagship EU-funded collaborative project TransMalariaBloc, which aimed to develop and test the efficacy of malaria transmission-blocking interventions. They included transmission-blocking vaccines and drugs/remedies, which although administered to humans function to stop the parasite in the vector or to compromise the vector itself, and engineering mosquitoes to become resistant to infections, by boosting their natural immune system or by supplying them with alternative resistance properties.

At the end of the meeting it was the general view that rapid progress has been made in the last decade in understanding the biology of the vectors and how they transmit pathogens. It was appreciated that this is largely due to the numerous technologies that were facilitated by the sequencing of the first mosquito genome in 2002. Indeed, vector research provides many avenues to interesting science that spans many diverse disciplines of modern biology. It was a central highlight of the meeting that multidisciplinary approaches dealing with vector-borne diseases as an integrated system can now provide a fuller understanding of disease transmission. At the same time, it was clear that the synthesis of knowledge and material generated over the years by the more traditional research approaches has generated an immense translational potential of vector research in public health, generating great enthusiasm among participants. What was also evident is that the distance between laboratory and field experimental research has decreased substantially and indeed the combination of these approaches was the central pillar in most of the works presented during the meeting. A third pillar is sophisticated informatics, which not only has become an indispensable routine tool but also has established itself as one of the frontiers in cutting-edge vector biology research. Yet, in the endeavor for translation, mathematical modelling is fundamental, and a few presentations in which modelling was either used to fit experimental data or generate new testable hypotheses started to appear and will surely do more so in the future. As roadmaps to translation and impact are established, sustainability and prevention will need to be discussed, whereby the impact of environmental and societal factors is expected to be central.

**Figure 2:**
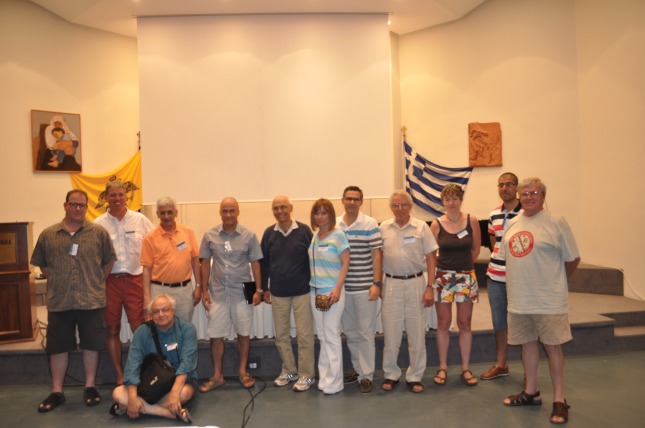
Contributors to the special symposium for Fotis C. Kafatos: From left to right: Larry Zwiebel, Anthony A. James, Kitsos Louis (sitting), Kostas Iatrou, George Dimopoulos, Fotis C. Kafatos, Dina Vlachou, George K. Christophides, Marcelo Jacobs-Lorena, Stephanie Blandin, Mike A. Osta and Barry Beaty.

Cutting edge molecular studies of disease vectors have been made possible because of the vision and thrust of few people. One of them is Fotis C. Kafatos, who retired after many years of serving science and science politics at the highest levels. The mosquito and vector communities honored Fotis and his contributions to the field with a special evening symposium. The session was chaired by Anthony A. James and comprised 10 speakers who were Fotis’s colleagues, collaborators or indeed members of his international laboratory spanning different periods of his distinguished career (Figure 2). Kostas Iatrou, one of Fotis’s most senior collaborators, began by providing an overview of Fotis’s career as a young Professor at Harvard, Director of the Institute of Molecular Biology and Biotechnology in Crete, Director General of the European Molecular Biology Laboratory, founding President of the European Scientific Council, and Professor of Immunogenomics at Imperial College London. The next talk described Fotis’s contribution to the field molecular biology of disease vectors based on support from the MacArthur Foundation in 1989. It was given by another MacArthur recruit, Marcelo Jacobs-Lorena. The fruit fly was first genetically transformed in 1982, but this advancement did not spill over into vector research until 1989 when the MacArthur foundation led by Jonas Salk recruited a number of prominent scientists to close this gap, including Fotis. A landmark meeting for this burgeoning group and other members of the scientific community was held in Tucson, Arizona in 1991.

Another member of the MacArthur network, Barry Beaty described the importance of establishing the Biology of Disease Vectors (BVD) course, which was made possible through the generous support of the WHO and TDR. The purpose of BDV was to attract young scientists to vector research and to train them using hands on laboratory methods. The course was held at Colorado State University every other year and at different international locations in alternating years such as Crete, Mali, Thailand, and England. Down time was built into the schedule of the course where participants could enjoy the location but also seek out local collaborators. Barry shared photos from various meetings, including Fotis teaching in the laboratory. He also shared some fascinating metrics showing dramatic increases in the number of high-level publications and NIH grants in the years after the initiation of the course, revealing the tremendous impact of the course on vector research. He concluded with a view shared by all participants that given its lasting legacy it is a shame that BVD no longer exists and it is the hope of the community that this great institution could be revived in the future. Like the Kolymbari meeting, BDV is one of the most important traditions in the field and therefore must be continued.

The chair of the session Anthony A. James gave the next talk. As another of the MacArthur recruits, he returned to a goal that was laid out by the network members at their meeting in Tucson, which was to genetically transform a mosquito vector. Accomplishing this goal took approximately 10 years. The main hurdle was that, although adopting robust genetic tools available in the fruit fly was generally useful, the routinely used Drosophila P-element was not a viable approach in mosquitoes; therefore, development of new technologies was needed. He showed original slides he presented from this period and the first pictures from a genetically modified *Ae. aegypti* mosquito whose eye color was modified by expression of a non-mosquito gene.

George Dimopoulos gave the first of a series of five talks that described interactions of Fotis with his laboratory members. During his years as a postdoc with Fotis at the EMBL, he cited Fotis’s rigor and ambition as qualities that made him a great mentor and scientist. He also touched on his efforts to distill and emulate Fotis’s leadership while running his own group and described Fotis as the type of scientist whose intellect made it possible to tread into new territory with confidence and excitement. These thoughts were echoed by Larry Zwiebel, and added that a charisma of Fotis that made him a great mentor is that he allowed his laboratory members to freely explore uncharted scientific territories, even outside his main scientific interests. His scientific curiosity, adventurous spirit, generosity, and distinctive ability to always see the glass as half full rather than half empty provided the inspiration required to forge ahead.

George Christophides, Fotis’s closest collaborator in the last 15 years and joint director of the Vector Immunogenomics laboratory at Imperial College London until Fotis’s retirement in September 2012, gave the presentation he gave at the very first Kolymbari meeting in 2003, when he was still a postdoc with Fotis at the EMBL. His talk put into context the innovations in genomics and functional genomics tools that the laboratory had made from the first EST and oligonucleotide arrays to the first full genome microarray that followed the *An. gambiae* genome sequencing. The inspired contributions of Fotis during that time had decisively transformed the field of vector molecular biology and opened the avenues for the great work that was presented in the Kolymbari meeting 10 years later. Stephanie Blandin who earned her doctorate degree in Fotis’s laboratory in EMBL presented how the functional genetic analysis in mosquitoes was pioneered by establishing a technique for gene silencing by RNA interference. It was a robust and straightforward method to achieve this in mosquitoes at a time when traditional gene knockout was not possible. The method was instrumental in proving that the mosquito immune system limits malaria parasites and is still in widespread use today. She finished by sharing a famous mantra Fotis used in the laboratory: ‘Think, do, publish’. Dovetailing nicely with her talk, Mike Osta described how, during his postdoctoral work with Fotis and in collaboration with George Christophides, he used that technique to define mosquito proteins that interact with malaria parasites. This was the first demonstration of genetic epistasis between genes that influence parasite infection. He concluded that those who have written a manuscript with Fotis know he is a meticulous writer, and told an anecdote about the comments and corrections he got on his first manuscript with him. The pages Fotis sent to him by fax were annotated with extensive hand-written corrections, covered with symbols and arrows, using every last bit of free whitespace and continuing on extra sheets of paper. Mike had to seek the help of senior members of the laboratory to help decipher them.

Kitsos Louis, a long-standing collaborator of Fotis, who had been the Kolymbari meeting organizer from the beginning until 2011, gave the final talk of the symposium starting with a photo of Fotis swimming in the sea in front of the Academy. He remembered that the fact that once he expressed an opinion about the quality of a computer in the lab and wrote a program to do DNA sequence analysis made Fotis to think he is a bioinformatician. This led him to work with Fotis and Michael Ashburner on the European Drosophila Genome project and in many other projects throughout the years, including AnoBase the ancestor of VectorBase. Fotis and he were never in each other’s laboratories but they have been on more than 40 papers together over the years.

Overall the session provided a fantastic overview of Fotis’s career as a leader, innovator, and mentor. Younger people in the audience who knew of Fotis but who had never worked or interacted with him got to appreciate the magnitude of the person and his contributions not only to the vector biology field but also to science as a whole. Quoting Barry Beaty, it was a great opportunity for everyone to thank Fotis for ‘illuminating the black box of vector biology’. Capping off this Gala night was a banquet under the stars, with traditional Cretan food and wine followed by music and dancing. It was another occasion for Fotis to display his leadership, this time on the dance floor where he danced all night long together with the other meeting participants. As Kitsos Louis said: ‘Ω*Π*A Φ*ώ*τη’.

